# Development and initial validation of questionnaire on predictors for the use of hearing protection devices among noise exposed manufacturing workers in Tanzania: A methodological study

**DOI:** 10.3389/fpubh.2023.1102343

**Published:** 2023-02-09

**Authors:** Israel Paul Nyarubeli, Alexander Mtemi Tungu, Ståle Pallesen, Bente Elisabeth Moen, Simon Henry David Mamuya

**Affiliations:** ^1^Department of Environmental and Occupational Health, Muhimbili University of Health and Allied Sciences, Dar es Salaam, Tanzania; ^2^Research Group for Occupational and Environmental Medicine, Department of Global Public and Primary Care, University of Bergen, Bergen, Norway; ^3^Department of Physiology, Muhimbili University of Health and Allied Sciences, Dar es Salaam, Tanzania; ^4^Department of Psychosocial Science, University of Bergen, Bergen, Norway; ^5^Centre for International Health, Department of Global Public and Primary Care, University of Bergen, Bergen, Norway

**Keywords:** hearing protection device, noise, manufacturing, workers, questionnaire, validity, reliability

## Abstract

**Introduction:**

The use of hearing protection devices (HPDs) has been an intervention of choice in many workplaces such as in the construction industry for quite some time due to impractical effects of engineering and administrative interventions. Questionnaires for assessment for HPDs use among construction workers have been developed and validated in developed countries. However, there is limited knowledge of the same, among manufacturing workers in developing countries that are assumed to have a different culture, work organizations and production processes.

**Methods:**

We conducted a stepwise methodological study to develop a questionnaire to predict the use of HPDs among noise exposed workers in manufacturing factories in Tanzania. The questionnaire included 24 items and was developed through rigorous and systematic procedures involving three steps; (i) item formulation that involved two experts, (ii) expert content review and item rating that involving eight experts with vast experience in the field, and (iii) a field pre-test that involved 30 randomly selected workers from a factory with similar characteristics as a planned study site. A modified Pender's Health Promotion Model was adopted in the questionnaire development. We analyzed the questionnaire in terms of content validity and item reliability.

**Results:**

The 24 items were categorized into seven domains i.e., perceived self-efficacy, perceived susceptibility, perceived benefits, perceived barriers, interpersonal influences, situational influences and safety climate. The score for content validity for each item was satisfactory as the content validity index ranged between 0.75 to 1.00 for clarity, relevance, and essentiality criteria. Similarly, the scores for the content validity ratio (for all items) were 0.93, 0.88 and 0.93 for clarity, relevance, and essentiality, respectively. In addition, the overall value for Cronbach's alpha was 0.92 with domain coefficients: perceived self-efficacy 0.75; perceived susceptibility 0.74; perceived benefits 0.86; perceived barriers 0.82; interpersonal influences 0.79; situational influences; 0.70; and safety climate 0.79. The mean inter-item correlation was 0.49 suggesting good internal consistency.

**Discussion and conclusion:**

The developed and preliminary validated questionnaire can be used to predict the HPDs use among noise exposed manufacturing factory workers. Future surveys using this questionnaires warranted for further validation of the scale developed.

## 1. Introduction

Developing countries are challenged with attaining and sustaining a descent work environment in the era of growing economy (industrialization) that are inherently characterized by increased number of workplaces with high noise levels especially in manufacturing industry (1, 2). The ultimate result for continuously working in high noise levels is development of temporary threshold shift of hearing (3), and noise-induced hearing loss (NIHL) (4–6). However, this can be avoided or rather prevented through implementation of a hierarchy of hazard controls, such as elimination, engineering, administrative or personal protection (in the order of decreasing effectiveness) (7). These measures include installing less noisy machines and or processes, automation of processes, installing muffler/silencers/sound absorbers to existing machines, increasing distance from the noise source to the worker, installing noise barriers/acoustic enclosure, job- rotations, re-schedule work time to reduce worker exposure, provision of noise-free quite room for breaks, and establishment of hearing conservation programmes with the use of hearing protection devices (HPDs) (7). However, the applicability of effective engineering measures has been infeasible or problematic in manufacturing factories operating in many developing countries including Tanzania due to the inherent nature of the existing machine-technology, nature of work (mostly manual work) and the costs attached to noise interventions (1). The remaining interim solution is the appropriate use of HPDs among noise exposed workers to minimize the risk for developing NIHL (8–10).

The use of HPDs at workplaces has been an intervention of choice in many workplaces such as in the construction industry for quite some time due to impracticability of engineering and administrative controls (10–14). Manufacturing factories in developing countries such as iron and steel factories have implemented the same intervention (15, 16). However, the use of HPDs is occasionally observed during workplace site visit and during pre-informed or planned legislative compliance follow-ups. The implementation of such intervention has been, in most cases, tied-up with behavioral models and theories such as the Health Promotion Model, the Health Belief Model, Theory of Planned Behavior and Theory of reasoned Action that can best predict human behavior toward the use of HPDs at work (17). Previously, we documented workers in manufacturing factories such as iron and steel working in high noise environment exceeding the occupational exposure limit of 85 dB(A) without using HPDs (1). These workers had high prevalence of NIHL (18). In a review of studies conducted within this sector in developing countries (19) we found that workers in most factories were not provided with HPDs and in few instances where HPDs were provided the use has very low and the prevalence of NIHL was high, suggesting existence of a behavioral and cultural gap (attitude and beliefs) that influence the desirable behavior (consistence use of HPDs).

Although various health behavior models have been established and tried out to predict human behavior with regard to the use of HPDs among noise exposed workers (17, 20–23), the purpose of each model has been to develop the most optimal tool that would best predict the likelihood of the use of HPDs among individual workers while working in high noise levels (17, 23), thereby establish and implement effective intervention on HPDs use at a particular workplace. However, it has been always problematic for the researchers to develop and align questionnaires between one existing model or theory with the existing environment in manufacturing factories (24). A combination of knowledge and skills of understanding the workplace environment, workers beliefs, experiences and perceptions attributed to the use of HPDs is critical to develop relevant instruments.

Researchers in the construction industry has for some years documented the pertinence of the Pender's Health Promotion Model and have recorded some modifications to this model across times (11, 12, 14, 25, 26). It is conceivable that the same model may be applied to the context of the manufacturing industry (13). As it applies to occupational noise and hearing loss perspective, this model explains the factors underlying motivations to positively influence and engage individual workers' health behavior in consistently and effectively use HPDs at work as a personal protective measure against NIHL (27). In addition, the model aims at maximizing benefits for HPDs use against the existing barriers (25). However, to the best of our knowledge, the modified Health Promotion Model has not received much attention among researchers in the context of workers in the manufacturing factory such as the iron and steel industry particularly in Tanzania. The working environment, manufacturing process, the work culture, norms and hence the behavior are likely to be different from other work environments such as the construction industry and consequently there is a need to develop and validate a new questionnaire suitable for this group of noise exposed workers (28).

To successfully and effectively implement a planned intervention targeting the use of HPDs among noise-exposed manufacturing factory workers in Tanzania, two things were a prerequisite. Firstly, the development of a complete tool or questionnaire for soliciting the predictors of HPDs use, and secondly to systematically validate the prepared questionnaire. Therefore, the purpose of this study was to develop and initially validate a questionnaire for predictors of HPDs use among noise-exposed manufacturing factory workers in Tanzania. During the process, pre-testing was indispensable to ensure that workers understand the prepared questionnaire and provide the needful information (29).

## 2. Materials and methods

### 2.1. Methodological design and respondents

This study was conducted within one iron and steel factory in Dar es Salaam. The factory had similar working environment to the ones intended for a planned intervention study. Respondents included in the current study were male workers who worked in factory and who were exposed to noise level above 85dB (A). Additionally, eight (8) experts with knowledge and skills within the occupational health and safety profession were involved in different stages of the questionnaire development.

### 2.2. Sample size estimation for pre-test

The estimation of sample size for the pre-test assumed that 10% percent of workers in the pre-tested factory were likely to encounter problems in understanding and hence answering items in the prepared questionnaire appropriately. To achieve a power of 90% to detect a problem present for one in ten participants, at a 95% confidence interval (2-sided), 30 workers were required (30).

### 2.3. Questionnaire development

The questionnaire was developed through the following stages.

#### 2.3.1. Theoretical framework

**Figure 1:**
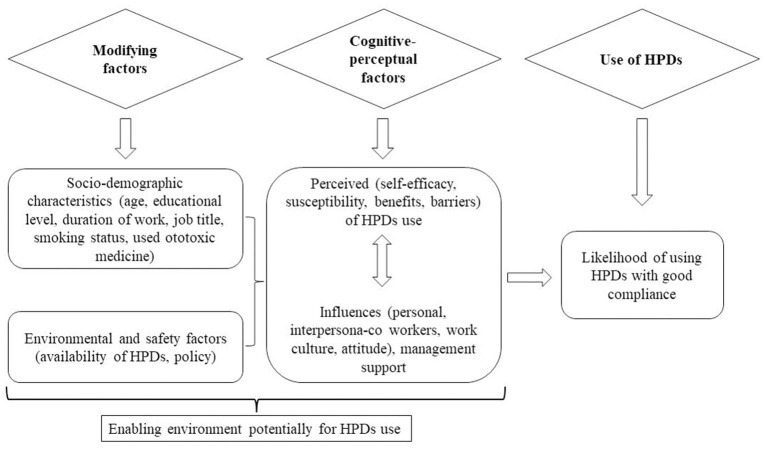
A schematic diagram of a modified Pender's health promotion model (HPM) used in development of questionnaire for predictors of Hearing protection devices use among noise exposed manufacturing workers in Tanzania.

In this study, we used the modified Health Promotion Model that explains predictors of health-related behavior toward HPDs use at noisy workplace which have been investigated in the construction industry (12). This model is based on the three fundamentals for behavior change that include: (i) individual characteristics and experiences such as the socio-demographic characteristics, interpersonal and situational influences, (ii) behavior-specific cognitions and affect such as perceived self-efficacy, susceptibility, benefits and barriers of using the HPDs, and (iii) the expected behavior outcome which, in this case, was the consistent and effective use of HPDs at work (27). We adopted and operationalized four cognitive domains of the model i.e., the perceived self-efficacy, perceived susceptibility, perceived benefit and perceived benefit to fit to the manufacturing factory as it was used in other industries (14, 20, 31) (Figure 1). In addition, we added three domains i.e., interpersonal influences, situation influences and safety climate as critical environmental or organizational predictors (ecological model) found to influence HPDs use at workplace (32) making a total of seven domains.

#### 2.3.2. Item formulation/development

**Figure 2:**
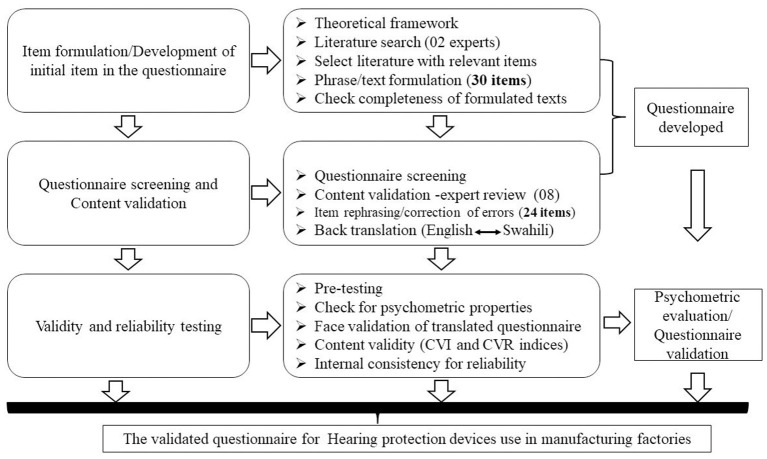
Schematic diagram showing stepwise development of hearing protection devices use among workers in manufacturing factories in Tanzania.

Two researchers embarked on constructing/formulating items that addressed predictors of HPDs use among noise exposed workers. We conducted literature reviews by searching relevant publications and survey tools available on the use of HPDs, predictors of wearing hearing protectors, factors for effectiveness of HPDs, interventions to reduce noise induced hearing loss and effectiveness of HPDs in noise exposed workers. This was conducted through online search engines such as PubMed-Medline, Google Scholar, and Embase. These engines were those assumed to contain most of the published materials (or indexed) regarding HPDs. Online search terms used were:-[(workplace or occupational^*^ or industr^*^ or factor^*^) AND (noise or sound^*^ or noise induced) AND (“hearing loss” or “hearing impairment”) AND (“hearing protect^*^” or earmuff or earplugs or “hearing protection devices”) AND (intervention or effectiveness or success^*^) AND (questionnaire^*^ or tool) AND (develop or construct or formulat^*^) AND (valid^*^ or reliabilit^*^) AND (benefit or barrier)]. We included published materials and articles with imbedded questionnaire texts on interventions targeting the effectiveness of HPDs use such as earmuffs or earplugs among noise exposed workers through health promotion model. The material consisted of published articles with Appendices or Supplementary material; those used in survey or validated among noise exposed workers; those written or translated into English Language; close ended questions; and questions with Likert scale responses. Inaccessible published materials such as articles, manuals or questionnaires were excluded. Furthermore, we visited some websites providing specific information on the use of HPDs in noise exposed workers such as the Cochrane Library (for systematic reviews on effectiveness of HPDs), the Health and Safety Executive (HSE-UK) and the National Institute for Occupational safety and Health (NIOSH) under the Centers for Disease Control and Prevention (CDC)—Searching for HPDs survey questionnaires, manuals and guide documents relevant for noise exposed workers in the manufacturing industry. In this process, only four articles met our screening criteria. Based on these four selected articles, we extracted and formulated a total of 30 items relevant for our selected theoretical framework. We arranged items and check for ambiguity of phases or words and the overall flow of questions (Figure 2).

### 2.4. Questionnaire initial screening

We screened the 30 formulated items and modified some of the phrases to fit the context of the manufacturing factories in developing countries including Tanzania. Such items were for example; *- “I will feel better if my workplace is less noisy”* (33) was modified to “I work better if my workplace is less noisy,” “*Wearing hearing protectors stops me from hearing what I want to hear”* was modified to “Hearing protectors makes it hard to communicate with co-workers” (33). At this point, we removed six items initially formulated as they were deemed irrelevant for the existing work environment or might be misunderstood/meaningless in the context of the existing work tradition. Example of such items included “It's easier to close the ear using the finger/hand rather than wearing an ear plug” (34) and those related to experiences of using HPDs. Also, we made a general modification of rephrasing most items and remove the original type of factory such as sawmill (34) to reflect the manufacturing factory we intended for the present purpose. We remained with 24 items after initial screening (Table 1).

**Table 1:** Items in the developed questionnaire before and after expert review process.

**Construct**	**No**	**Text before expert review**	**Text after expert review**	**Item code**
Perceived self- efficacy (SE)	1	I can tell when I need to wear my hearing protection (12, 33)	I can tell when I need to wear my hearing protection devices	SE1
	2	I am sure I can ask for help if I have a hard time wearing protection (12, 32)	I can ask If I need help on how to wear my hearing protection	SE2
	3	I think preventing hearing loss from noise is very important to my supervisor (32)	I can protect myself from noise-induced hearing loss	SE3
Perceived susceptibility (SS)	4^*^	My hearing will not be affected by noise, even if I don't wear hearing protection (12, 33, 34)	My hearing won't be affected by noise at work, even if I don't wear hearing protection	SS1
	5	I believe exposure to loud noise can hurt my hearing (12, 34)	Exposure to high noise levels can hurt my hearing	SS2
	6^*^	It wouldn't be a big problem for me if I lost some of my hearing (12, 33, 34)	It wouldn't be a big problem for me if I lost some of my hearing	SS3
Perceived benefits (BEN)	7	Preventing hearing loss is very important to me (12, 34)	Preventing hearing loss is very important to me	BEN1
	8	Wearing hearing protection protects me against hearing loss from noise (12, 32)	Wearing hearing protection devices protects me against hearing loss from noise	BEN2
	9	I work better if my workplace is less noisy (33)	I work better if my workplace is less noisy	BEN3
	10	Wearing hearing protection keeps me out of annoyance from noise (34)	Wearing hearing protection keeps me out from annoyance caused by loud sound	BEN4
Perceived barriers (BAR)	11^*^	Hearing protectors stop me from hearing what I want to hear (33)	Hearing protectors makes it hard to communicate to co-workers	BAR1
	12^*^	It takes too much time to use hearing protection (12, 32)	It takes too much time to get used to wearing hearing protection devices	BAR2
	13^*^	Wearing hearing protection is unsafe because it blocks out danger signals (32)	Wearing hearing protection devices is unsafe because it blocks out danger signals	BAR3
	14^*^	Hearing protectors are too uncomfortable for me to wear (12, 32, 33)	Wearing hearing protectors is uncomfortable for me	BAR4
Interpersonal influences (INF)	15	Other workers at this site reminds me when I need to wear hearing protectors (12, 32)	Other workers at this site reminds me when I need to wear hearing protectors	INF1
	16^*^	Other workers at this site make fun of me when I wear hearing protection (12, 32)	Other workers at this site make fun of me when I wear hearing protection devices	INF2
Situation influences (SINF)	17	I can choose from several types of hearing protectors from this site (12, 32)	There are several types of hearing protection devices that I can choose from in this work site	SINF1
	18	My supervisor thinks I need to wear hearing protection, even when my noise is short. (12, 32)	My supervisor thinks I need to wear hearing protection, even when the noise is low	SINF2
	19	It is our factory rule that I use hearing protection while working in noisy environment (12, 32)	It is our factory rule that I use hearing protection while working in noisy environment	SINF3
	20	My supervisor sets a good example for me when it comes to hearing protection (12, 32)	My supervisor sets a good example for me when it comes to the use of hearing protection devices at work	SINF4
Safety climate (SCL)	21	My supervisor frequently checks to see if I am obeying the safety rules (12, 32)	My supervisor frequently checks to see if I am obeying the safety rules regarding wearing hearing protectors	SCL1
	22	My supervisor talks with me about how to improve safety (12, 32)	My supervisor talks with me about how to improve safety	SCL2
	23	My supervisor reminds me to work safely if I am not doing so (12, 32)	My supervisor reminds me to work safely if I am not doing so	SCL3
	24	My supervisor says I must wear my hearing protectors, even if they are not comfortable. (12, 32)	My supervisor encourages me to wear hearing protection devices, even if they are uncomfortable.	SCL4

* Items with negative statements.

Numbers in parentheses next to the question shows the reference(s) from which the original item was extracted.

The 24 developed and screened-items were aligned into seven domains pre-identified in our theoretical framework. We adopted somewhat similar item operationalization and grouping that were used in construction industry (12, 14, 20). The grouping was performed by two researchers. Briefly, the items that deemed to display a characteristic of a particular domain were grouped together. For example, four items predicted beliefs in the positive results of perceived action (gained value or benefit for perceived use of HPDs) and were consequently grouped into perceived benefits; four items that displayed perceived challenges or impediments in HPDs use were grouped as perceived barriers domain and so on. The seven domains of the questionnaire therefore consisted of perceived efficacy = 3 items; perceived susceptibility = 3 items; perceived benefits = 4 items; perceived barriers = 4 items; interpersonal influences = 2 items; situational influences = 4 items; and safety climate = 4 items (Table 1).

We additionally equipped our questionnaire with individual (modifying) factors that have been found to influence HPDs use at workplace (14, 17, 20, 26, 32, 35, 36). Such factors include socio-demographic factors that formed a separate dimension. The aim of this was to enable the questionnaire to collect basic information about the individual workers and their working environment. Such items were, worker's age, job title or task performed, educational level, duration of work, previous noise exposure, smoking status, the use of ototoxic medicine, engagement into any leisure activities after work and availability of HPDs at his/her workplace.

A five-point Likert scale [psychometric scale consisting of multiple responses used in systematic evaluation of reliability and validity (37)] was then used as the response alternatives to indicate the degree of agreement with each statement. Literature has documented that responses to scale together with its validity are to some extent influenced by the number of alternatives available i.e., scale width (38). Scale width of less than four points might result in underestimated alpha coefficients for items (39). Likert-scales have been widely used in psychometric research (40–42). In addition, the Likert scale has been found to be used to measure many types of affective characteristics and produce high internally consistent data as one of its advantages (43). The five-point Likert scale we chose ranged from *strongly disagree, disagree, neutral, agree and strongly agree*. We assumed that workers with overall positive behavior toward the use of HPDs would tend to agree with positive worded items and disagree with negative worded items.

### 2.5. Expert review

The purpose was further to assess and evaluate the extent to which the constructed questionnaire items were representative of the entire domain in which the main study was intended (44–46). By involving expertise in the process of development and validation of questionnaires, one can discover and rectify problems within questionnaires and modify or rephrase items and questions that are found to be problematic (47). We therefore sent a prepared questionnaire to eight different experts in the field with vast experiences in terms of occupational health and safety separately in order to evaluate the items. We prepared a signed letter with instructions on how to carry out the evaluation. We enquired them to: (i) identify problems within each item (phrase) that was likely to be misunderstood, (ii) assess and identify items that were unclear and problematic (iii) review, rate and provide expert opinion on the quality and clarity, relevance and essentiality of the questionnaires and add open commentaries (if any) (48). After this process, we received experts' feedbacks and opinions that helped in rephrasing and shape the developed questionnaire as summarized in Table 1.

### 2.6. Questionnaire translation

Originally, we developed the questionnaire items in English. However, our target study population were workers in manufacturing factories who were primarily Swahili language speakers and translation of the questionnaire was therefore necessary. The questionnaire was therefore translated into Swahili and thereafter back-translated into English. The backtranslation was carried out by an independent bilingual researcher who did not take part in the original translation. The two English versions were compared for clarity and results was satisfactory (49).

### 2.7. Pre-testing

We conducted the pre-testing of the completed questionnaire among 30 factory workers. The factory next to our planned intervention site (3) allowed 30 of their workers to participate. This factory had similar working environment including end-product manufacturing processes to our planned intervention site. In all, 30 randomly selected workers were invited to participate and all consented. We explained the purpose for this pre-test to the participants and assured them of the confidentiality of information gathered. Data on demographic characteristics such as their age, job title, education level, duration of employment, previous noise exposure, smoking status, and the use of HPDs were collected.

### 2.8. Time to administer the questionnaire

In a pre-test, we recorded the time (in minutes), an individual respondent used to answer the set of items in the prepared questionnaire. This was necessary as it may affect the quality of information collected which was assumed to be contributed by the length of questions themselves, the clarity of language used, the comprehension and relevance of questions to the study environment and the study population (36). An average of 30 min is by some literature, regarded as optimal to maintain the attention of respondents (50). We selected places (rooms or chambers used during breaks or rest periods) within the factory in which individual worker felt comfortable and undisturbed or uninfluenced by surrounding environment while answering the questionnaire.

### 2.9. Ethical consideration and informed consent

This paper is part of the project “*Intervention to reduce an occupational noise exposure by using earmuffs and earplugs in factory workers in Tanzania*.” We have obtained ethical clearance from both the Regional Committee of Medical and Health Research Ethics (REK-VEST) in Norway and from the Muhimbili University of Health and Allied Sciences (MUHAS) Ethics Committee in Tanzania. Individual workers who participated in the pre-test were contacted and informed about the pre-test activities to be conducted and provided their written consent. Information collected were treated with confidentiality.

### 2.10. Statistical analysis

Information gathered in the development of this questionnaire and pre-test thereof were presented in various ways. First, information gathered from the expert review process of the questionnaire development, was analyzed and computed on spreadsheets using Microsoft Excel, (available at: https://office.microsoft.com/excel). Secondly, measures of questionnaire reliability and pre-testing were performed using IBM SPSS Statistics for Windows, Version 27.0. Armonk, NY: IBM Corp. Responses on the Likert scale were coded and assigned numerical values ranging from 1 = strongly disagree to 5 = strongly agree. Negatively worded questions were reversed before the analysis.

#### 2.10.1. Content validity

To theoretically analyze the adequacy with the items in terms of representativeness of each domain, we calculated the content validity indices (CVIs) and content validity ratio (CVR) for both the individual items and the entire scale respectively (24, 51, 52). We used the ratings and scores obtained from the eight experts (53). Previously, we inquired each expert (among other things) to evaluate, and rate items based on three criteria i.e., clarity (1 = not clear, 2 = need revision, 3 = quite clear); relevance (1 = not relevant, 2 = need revision, 3 = relevant); and essentiality (1 = not essential, 2 = need revision, 3 = essential). During analysis, we merged the total scores into either 0 (denoting disagreement among experts) or 1 (agreement among experts). Individual items that achieved a total rating of 1 and 2 were coded as 0 and those rated 3 were coded as 1. CVR was calculated using the formula: CVR = (Ne-N/2)/(N/2), where *Ne*” is the number of expert rating 3- quite clear/relevant/essential” and *N*” is the total number of experts (52). CVI was computed as the means of CVR values of items in the scale. Microsoft Excel Spreadsheet (Microsoft Corporation, available at: https://office.microsoft.com/excel) was used for computation of CVR and CVIs. An CVI of 0.75 or higher is considered evidence of good content validity according to Lawshe's formula (52, 54).

#### 2.10.2. Reliability of the questionnaire

We used Cronbach's alpha as the measure for questionnaire reliability i.e., the degree to which the items in the scale reflect the same construct and thus relates to their sum score (inter-relatedness if the items within the test) (55). We computed alpha coefficient for each of the seven domains to assess within-construct item agreement and for the total questionnaire (55). We regarded an alpha coefficient of 0.70 as an acceptable threshold for scale reliability (56). We examined the internal consistency of each domain (the general agreement between multiple items that make up a composite score of the domain) by computing the mean inter-item correlation (57) considering the optimal inter-item correlation to be in the ranges of 0.15 to 0.50 (58).

## 3. Results

### 3.1. Socio-demographic characteristics of pre-test participants

During pre-test, our questionnaire was administered to a total of 30 workers. These workers had a mean age of 29 (SD = 5; range 22–38) years and had worked for 4 (SD = 4) years. The majority of the workers had primary education (70%). Only 7% of workers reported to have access to HPDs to use at work and about 17% attended leisure activities after work (Table 2). The socio- demographic characteristics of this sample resembles somewhat the population intended for the planned interventional study (3).

**Table 2:** Socio-demographic characteristics of the pre-tested sample of noise exposed workers in manufacturing factories in Tanzania (*N* = 30).

**Demographic variables**	**Frequency**
**Number, %**	
**Age (years)** Mean, SD 29 (5)	
**Duration of work (years)** Mean, SD 4 (4)	
**Job group/title**	
Tongsmen	4(13.3)
Cutters	7(23.3)
Pushers	5(16.7)
Foundry	7(23.3)
Melters	4(13.3)
Technician	3(10.0)
**Educational level**	
Primary	21 (70.0)
Secondary	6(20.0)
Tertiary	3(10.0)
**Current smoking**	
Yes	9 (30.0)
No	21(70.0)
**Ever used ototoxic medicine**	
Yes	2 (6.7)
No	28 (93.3)
**Engaged in leisure activities**	
Yes	5 (16.7)
No	25 (83.3)
**Have hearing protection device to use at work**	
Yes	2 (6.7)
No	28 (93.3)

### 3.2. Content validity of the questionnaire

Screening and refining of the HPDs use among workers in manufacturing factory questionnaire ended up with 24 items. The score for CVR for individual items ranged between 0.75 to 1.00 (7 or 8 expert rated the item as quite clear/relevant/essential). In addition, the CVI yielded a satisfactory overall score of 0.88, 0.93, and 0.90 for the clarity, relevance and the essentiality criteria, respectively (Table 3).

**Table 3:** Scores for Content Validity Index (CVI) and Content Validity ratio (CVR) for items in the HPDs use questionnaire among noise exposed workers in manufacturing factory.

	**Content validity ratio (CVR) with rating criteria score**		
**Item in a questionnaire**	**Clarity**	**Relevance**	**Essentiality**
SE1: I can tell when I need to wear my hearing protection devices	0.75	1.00	0.75
SE2: I can ask If I need help on how to wear my hearing protection	0.75	0.75	1.00
SE3: I can protect myself from noise-induced hearing loss	1.00	1.00	1.00
SS1: My hearing won't be affected by noise at work, even if I don't wear hearing protection	0.75	1.00	0.75
SS2: Exposure to high noise levels can hurt my hearing	1.00	0.75	0.75
SS3: It wouldn't be a big problem for me if I lost some of my hearing	1.00	1.00	1.00
BEN1: Preventing hearing loss is very important to me	0.75	1.00	0.75
BEN2: Wearing hearing protection devices protects me against hearing loss from noise	1.00	1.00	1.00
BEN3: I work better if my workplace is less noisy	0.75	0.75	1.00
BEN4: Wearing hearing protection keeps me out from annoyance caused by loud sound	1.00	1.00	1.00
BAR1: Hearing protectors makes it hard to communicate to co-workers	1.00	1.00	0.75
BAR2: It takes too much time to get used to wearing hearing protection devices	0.75	1.00	1.00
BAR3: Wearing hearing protection devices is unsafe because it blocks out danger signals	0.75	0.75	0.75
BAR4: Wearing hearing protectors is uncomfortable for me	1.00	1.00	1.00
INF1: Other workers at this site reminds me when I need to wear hearing protectors	0.75	1.00	0.75
INF2: Other workers at this site make fun of me when I wear hearing protection devices	1.00	1.00	1.00
SINF1: There are several types of hearing protection devices that I can choose from in this work site	0.75	0.75	1.00
SINF2: My supervisor thinks I need to wear hearing protection, even when the noise is low	1.00	1.00	0.75
SINF3: It is our factory rule that I use hearing protection while working in noisy environment	0.75	1.00	1.00
SINF4: My supervisor sets a good example for me when it comes to the use of hearing protection devices at work	0.75	0.75	1.00
SCL1: My supervisor frequently checks to see if I am obeying the safety rules regarding wearing hearing protectors	1.00	1.00	1.00
SCL2: My supervisor talks with me about how to improve safety	0.75	0.75	0.75
SCL3: My supervisor reminds me to work safely if I am not doing so	1.00	1.00	1.00
SCL4: My supervisor encourages me to wear hearing protection devices, even if they are uncomfortable.	1.00	1.00	0.75
^*^ *Content validity index (CVI)*	**0.88**	**0.93**	**0.90**

* CRITERIA: CVR(Critical) for a panel size (*N*) of 8 is 0.75.

### 3.3. Questionnaire reliability

**Figure 3:**
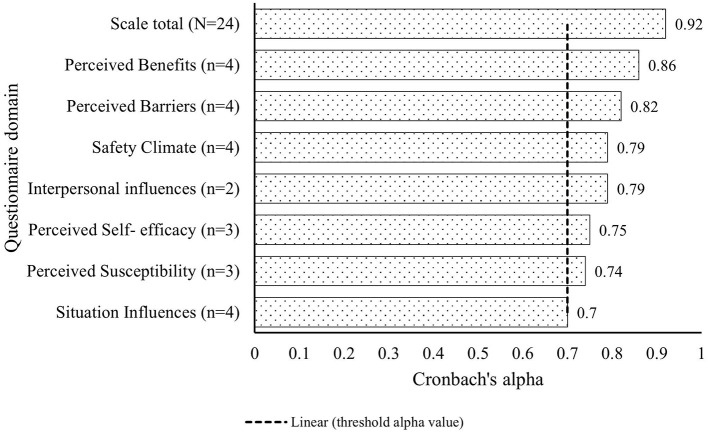
Internal consistency of the items in the seven domains of the use of hearing protection devices questionnaire measured by Cronbach's alpha coefficient. The vertical dotted line shows the threshold value of alpha coefficient for each domain and for the scale.

The 24-items in the final questionnaire had an overall internal consistency estimated using Cronbach's alpha coefficient (α) 0.92 (Figure 3). The mean inter-item correlation for all items in the questionnaire was 0.49. All the seven domains of the questionnaire scored satisfactory results in terms of alpha (Figure 3).

### 3.4. Time to complete answering the questionnaire

The average time taken by individual worker to complete answering the prepared questionnaire was 27 (SD = 3) min. This time ranged between 23 to 35 min.

## 4. Discussion

The main purpose of this study was to develop and preliminarily validate the questionnaire about predictors for HPDs use among manufacturing factory workers in Tanzania. We managed to develop the questionnaire with 24 items divided into seven domains. The psychometric assessment of this questionnaire during pre-testing yielded a suitable score for all items measured for content validity and reliability suggesting that it can be in use in surveys targeting HPDs use among noise exposed manufacturing workers. To our knowledge, this is probably among the first attempts to develop and validate the custom- suited and industry- specific questionnaire for soliciting predictors of HPDs use among noise exposed manufacturing factory workers in the Tanzanian context.

In our pre-test, items within the developed questionnaire showed satisfactory results for the CVR and CVI indices demonstrating good content validity. This was necessary as a planned survey among noise exposed manufacturing factory workers needs to have an appropriate data collection questionnaire for assessment and analysis of relevant cognitive construct and population characteristics regarding HPDs use (44). The systematic process for the development of the questionnaire involved a team of expertise, followed with field pre-test and provided evidence of content validity, representativeness and clarity of items that added to the reliability aspect (48). The experts involved in the present study had wide experience of practice and research in this field working in different countries and who also added advantage of knowing the intended project area and the population. Thus, a high degree of agreements among experts (CVR and CVI) in terms of the questionnaire appraisal phase suggested a good content validity, minimizing the likelihood of the bias in the developed questionnaire.

Contrary to the occupational safety and health law, most workers in our pre-test sample did not use HPDs which was the outcome behavior of interest analogous with some other factories of this kind (19). This was presumably due to unavailability and was documented in our previous observations among noise exposed workers in iron and steel factories (18). Nevertheless, the workers in these factories displayed a positive attitude toward the use of HPDs (59) which can be interpreted as good indicator for the use of HPDs especially when introduced within a framework of a modified HPM (32). It is worth noting that, the scope of the current work did not facilitate computation of predictive validity or establish the degree of correspondence (reported use vs. observed use) of HPDs use among noise exposed manufacturing workers. Yet, it might be an option when the planned survey is conducted.

Workers in our pre-test sample were able to complete the prepared questionnaire within an average of 27 min presumably due to short, relevant and clear items. This average time was somewhat shorter than the documented and hence, the suggested optimal time by other researchers of about 30 min (50) suggesting that workers presumably won't lose interest during answering survey questions. Hence, it is likely that, our developed questionnaire will be able to collect quality and reliable data in surveys while maintaining high response rate among noise exposed manufacturing workers (60). Furthermore, majority of the participants had primary education. This level of education in Tanzania refers to a complete education level of seven grades (excluding 3 years of pre- education). Such candidates are deemed able to read, write and comprehend various concepts. The current group of participants is in this realm similar to a large number of industrial working population in Tanzania and many other developing countries. Therefore, we believe that participants having primary education could reliably answer the questions and that similar results could be obtained in other factories.

The strength of this study includes the use of a rigorous and empirical procedure in the development and pre-testing of items in the questionnaire. The stepwise and combined process, harnessing expertise rating (knowledge, experiences and items evaluation) and field pre-test facilitated building a well refined yet, valid questionnaire (24). The current questionnaire reflects the real-working environment, work culture, traditions and accommodates technological situation currently existing in the manufacturing factories in developing countries which differs from those published mainly in the construction industry in the developed countries. However, it is important to acknowledge some limitations. Firstly, this questionnaire was developed and validated among male workers in the manufacturing factories in Tanzania. It might not be relevant for the general workplaces considering the complexity and differences in the nature of work, the culture, traditions or the type of industry. However, this questionnaire is useful and valid in the unique working environment. In these factories men are mostly employed due to the perceived nature of work in line with existing traditions. It might thus be a good idea to customize and pre-test the questionnaire in different work environments or in different work populations. Secondly, because the presented results reflect the initial stages of scale validation, some statistical analyses such as construct validity (e.g., predictive and convergent validity), confirmatory factor analysis, and test-retest reliability analysis were not conducted. A survey to assess HPD use among noise exposed workers in manufacturing factory is planned to yield data for further scale validation procedures. Lastly, although calculations of internal consistency (alpha) may be conducted on sample sizes as small as 30, provided high inter-correlations between the items (61), we still recommend the reliability of the scale to be investigated in lager samples in the future.

## 5. Conclusion

This study demonstrated the development and initial validation of the 24- item questionnaire to predict HPDs use among noise exposed workers in the manufacturing factories. Furthermore, more surveys among noise exposed manufacturing workers are warranted to further validate the scale.

## Data availability statement

The raw data supporting the conclusions of this article will be made available by the authors, without undue reservation.

## Ethics statement

The studies involving human participants were reviewed and approved by Regional Committee of Medical and Health Research Ethics-REK West-Norway and Muhimbili University of Health and Allied Sciences Ethics Committee in Tanzania. The patients/participants provided their written informed consent to participate in this study.

## Author contributions

IN: conceptualization, methodology, investigation, software formal analysis, data curation, writing—original draft preparation, writing—review, editing, resources, and fund acquisition. AT: conceptualization, methodology, investigation, data curation, writing—original draft preparation, and writing—review. SP: methodology, writing—original draft—review, and editing. SM: conceptualization, methodology, original draft review, project administration, supervision, and fund acquisition. BEM: methodology, investigation, writing, review, discussions, and supervision. All authors contributed to the article and approved the submitted version.
